# Brownian Motion Governs the Plasmonic Enhancement of Colloidal Upconverting Nanoparticles

**DOI:** 10.1021/acs.nanolett.4c00379

**Published:** 2024-03-18

**Authors:** Fengchan Zhang, Pedro Ramon Almeida Oiticica, Jaime Abad-Arredondo, Marylyn Setsuko Arai, Osvaldo N. Oliveira, Daniel Jaque, Antonio I. Fernandez Dominguez, Andrea Simone Stucchi de Camargo, Patricia Haro-González

**Affiliations:** † Nanomaterials for Bioimaging Group (nanoBIG), Departamento de Física de Materiales, Facultad de Ciencias, Universidad Autónoma de Madrid, Madrid 28049, Spain; ‡ Instituto Nicolás Cabrera, Facultad de Ciencias, Universidad Autónoma de Madrid, Madrid 28049, Spain; § São Carlos Institute of Physics, University of São Paulo (USP), 13566-590 São Carlos, São Paulo, Brazil; ∥ Departamento de Física Teórica de la Materia Condensada and Condensed Matter Physics Center (IFIMAC), Facultad de Ciencias, Universidad Autónoma de Madrid, E28049 Madrid, Spain; ⊥ Institute for Advanced Research in Chemical Sciences, Facultad de Ciencias, Universidad Autónoma de Madrid, 28049 Madrid, Spain; # Federal Institute for Materials Research and Testing (BAM), Berlin 12489, Germany; ∇ Friedrich Schiller University (FSU), Jena 07737, Germany

**Keywords:** upconversion, plasmon enhancement, optical tweezers, Brownian motion, nanoparticles

## Abstract

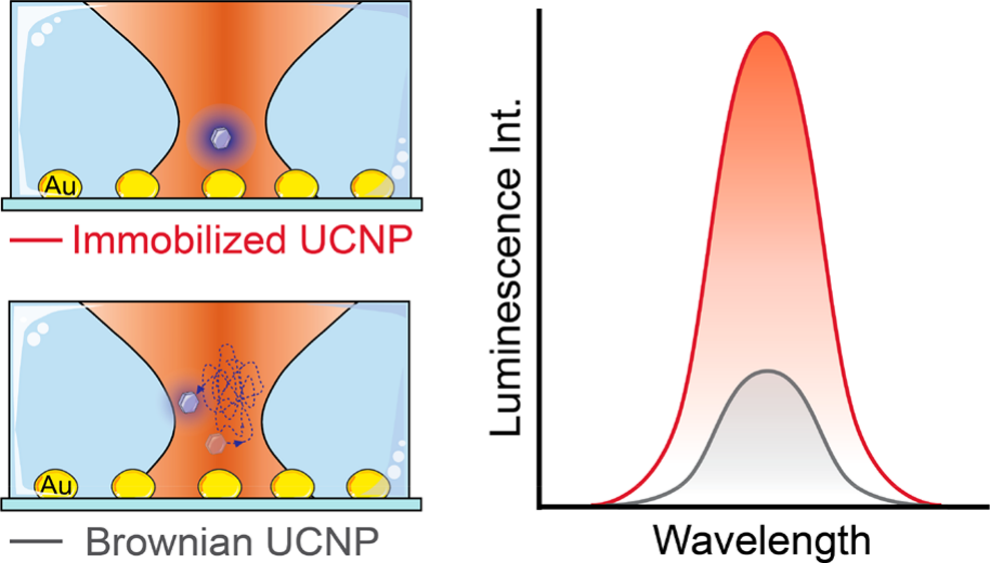

Upconverting nanoparticles are essential in modern photonics due to their ability to convert infrared light to visible light. Despite their significance, they exhibit limited brightness, a key drawback that can be addressed by combining them with plasmonic nanoparticles. Plasmon-enhanced upconversion has been widely demonstrated in dry environments, where upconverting nanoparticles are immobilized, but constitutes a challenge in liquid media where Brownian motion competes against immobilization. This study employs optical tweezers for the three-dimensional manipulation of an individual upconverting nanoparticle, enabling the exploration of plasmon-enhanced upconversion luminescence in water. Contrary to expectation, experiments reveal a long-range (micrometer scale) and moderate (20%) enhancement in upconversion luminescence due to the plasmonic resonances of gold nanostructures. Comparison between experiments and numerical simulations evidences the key role of Brownian motion. It is demonstrated how the three-dimensional Brownian fluctuations of the upconverting nanoparticle lead to an “average effect” that explains the magnitude and spatial extension of luminescence enhancement.

The unique ability of lanthanide-based upconverting nanoparticles (UCNPs) to convert infrared light into visible light makes them building blocks of modern photonics. UCNPs have been used to enhance the efficiency of solar panels,^1,2^ in healthcare,^3−5^ and for high-resolution bioimaging^6−8^ and remote sensing.^9−11^ Their main drawback is a low brightness, owing to the relatively low absorption coefficient of the lanthanide ions and their reduced quantum yield. The brightness of UCNPs has been enhanced by core/shell architectures that minimize nonradiative losses.^12,13^ Also, the absorption efficiency can be improved by doping engineering^14−16^ and combining UCNPs with plasmonic nanostructures (PNSs).^17−20^ The overlap between the plasmon resonance of PNSs and the absorption or emission bands of UCNPs results in enhanced excitation and radiative emission efficiencies, respectively. By optimizing the PNSs or controlling the UCNP–PNS distance, a critical parameter in plasmon-enhanced luminescence, brightness has been improved by up to 2–3 orders of magnitude.^21−26^ These improvements were demonstrated in static conditions where the UCNP–PNS distance is controlled and fixed^27−32^ Nevertheless, the situation becomes more complex if UCNPs are suspended in an aqueous medium. Brownian motion can introduce continuous fluctuations in the UCNP–PNS distance, competing with plasmon-enhanced luminescence. While plasmon-enhanced upconversion has been showcased in liquid media,^33,34^ the potential of using PNSs to improve the brightness of a single colloidal UCNP subjected to Brownian motion remains to be demonstrated. To explore plasmon-enhanced luminescence in a colloidal UCNP, 3D positioning of the UCNP in the proximity of the PNS is required. This manipulation should be contactless to minimally perturb the luminescence properties and Brownian dynamics. Thus, conventional methods, such as tip-assisted manipulation, are ineffective.

Optical tweezers (OTs) are a unique tool for contactless three-dimensional manipulation of individual UCNP in liquid media, allowing single UCNP spectroscopic studies or single-particle sensing in living cells.^35,36^ In this work, we use OTs to investigate the plasmon-induced brightness enhancement in a colloidal UCNP subjected to three-dimensional Brownian motion. The magnitude of plasmon-enhanced luminescence in a single UCNP as a function of OT–PNS distance was studied. Comparison between experimental data and simulations shows the role of Brownian motion in limiting the plasmon-enhanced luminescence of a colloidal UCNP.

Optical tweezing of individual UCNPs in the proximity of Au plasmonic nanoparticles (PNPs) is achieved by using a single-beam experimental setup (Figure 1a). A continuous-wave 980 nm laser beam is focused within a microchamber (13 mm diameter, 0.12 mm thickness) containing the colloidal dispersion of UCNPs by using an oil-immersion objective (100×/NA 1.4). The laser creates the OT and excites the upconverting luminescence. The UCNPs are hexagonal NaYF_4_:Tm^3+^,Yb^3+^ nanoparticles with an average size of 43 ± 4 nm (Figure 1b and c, synthesis method provided in section S1, Supporting Information). The 980 nm laser radiation is absorbed by Yb^3+^ ions, which transfer their energy to nearby Tm^3+^ ions, ultimately leading to visible emission (see Figure 1d). The OT system is coupled to a charge-coupled device (CCD) camera to visualize the incorporation of UCNPs into the trap by a real-time fluorescence image. The background contribution of the laser is blocked by two short-pass filters to record only the visible luminescence emitted by UCNPs. The microchamber is placed on a motorized stage for scanning the OT in both horizontal and vertical directions. The base of the microchamber is a glass substrate deposited with Au PNPs. Details about the experimental procedures for the deposition of Au PNPs are given in section S2, Supporting Information. Plasmonic enhancement can be induced by overlapping the surface plasmon resonance wavelength of the PNPs (λ_SPR_) with either the excitation or the emission wavelength of the UCNP. These two possibilities were explored by using Au PNPs of different diameters leading to plasmonic resonances at, approximately, 980 and 574 nm (see Figure 2a, b, c and e, f, g, respectively). In the first case (264 nm Au PNPs, λ_SPR_ ≅ 980 nm, Figure 2c), a local enhancement of the laser excitation efficiency is expected. In the second case (95 nm Au PNPs, λ_SPR_ ≅ 574 nm, Figure 2g), the plasmonic extinction mainly overlaps with the UCNP emission, and the Purcell effect is expected to be dominant. The broad plasmonic band also leads to a residual overlap with the 980 nm excitation radiation (see the gray dashed line in Figure 2g).

**Figure 1:**
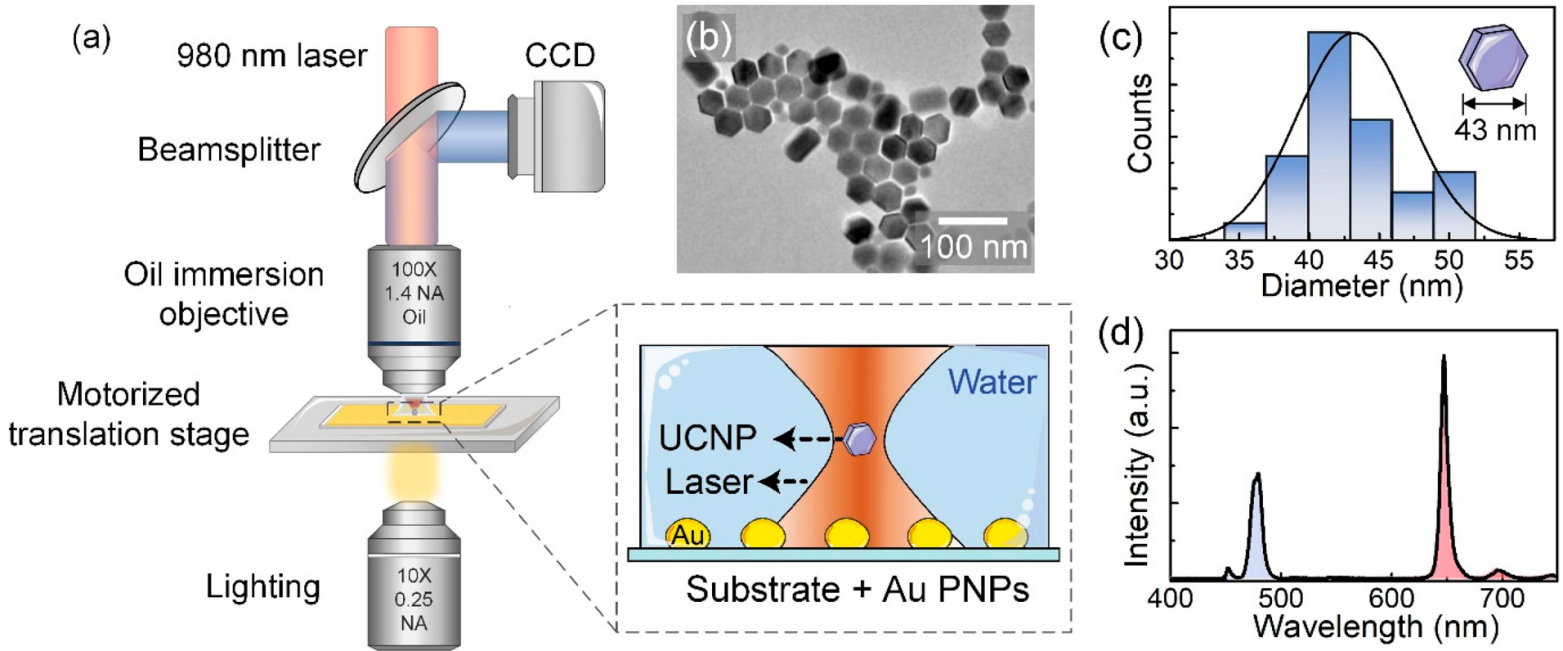
(a) Experimental setup used for optical tweezing of a single, colloidal UCNP in the presence of PNPs. (b) Transmission electron microscopy (TEM) image of the UCNPs. (c) Histogram of the size distribution of UCNPs as obtained from the analysis of TEM images. (d) Emission spectrum of the UCNPs under 980 nm excitation.

**Figure 2:**
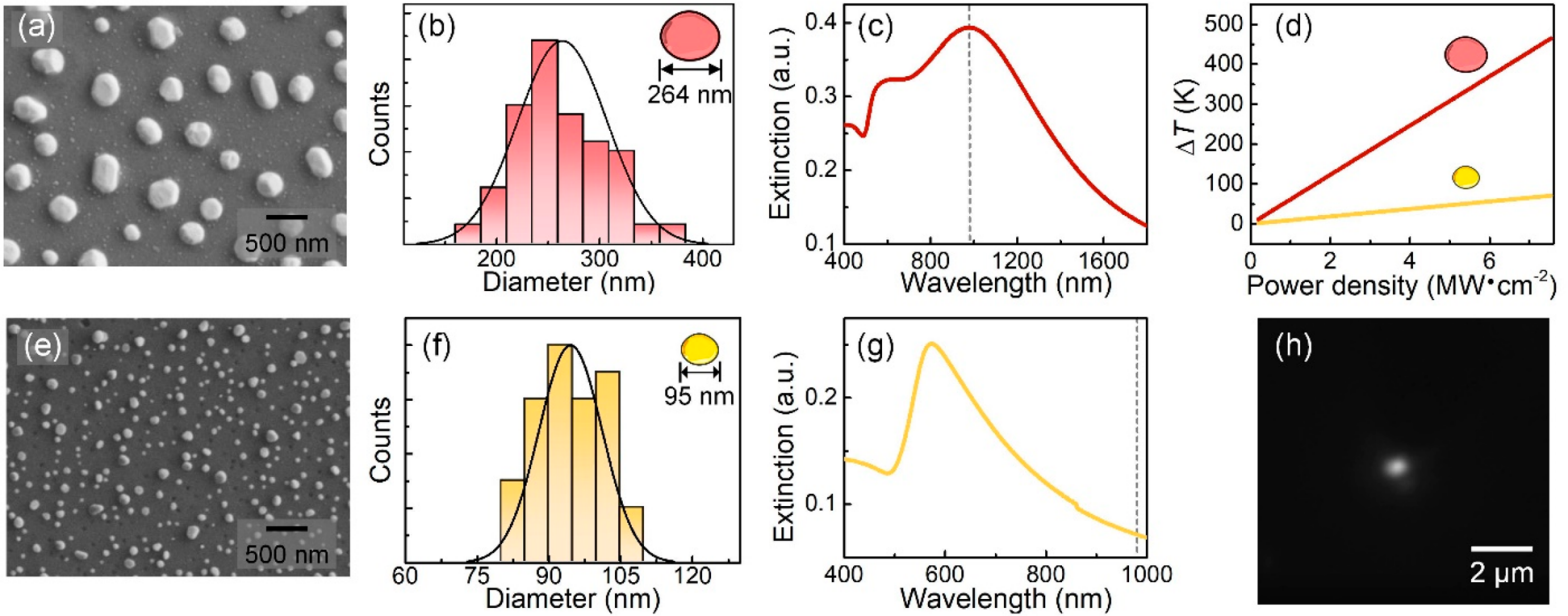
Scanning electron microscope images of the PNPs used in this work, with plasmon resonances at 980 and 574 nm (a and e, respectively). Histogram of the size distribution of Au PNPs with plasmon resonances at 980 and 574 nm (b and f, respectively). Extinction spectra of the PNPs showed the plasmon resonances at 980 and 574 nm (c and g, respectively). The gray dashed line indicates the wavelength of the trapping laser (980 nm). (d) Increase in local temperature induced by the PNPs under 980 nm irradiation as a function of the laser power density (red and yellow lines for the Au PNPs with plasmon resonances at 980 and 574 nm, respectively). (h) Fluorescence image of a single optically trapped UCNP on top of the substrate with PNPs with λ_SPR_ ≅ 574 nm under 980 nm excitation.

To study the possible plasmon-enhanced upconversion via a local increase of excitation efficiency (980 nm), we first tried to tweeze a single UCNP in the proximity of Au PNPs with λ_SPR_ ≅ 980 nm. No stable tweezing of the UCNP was achieved. We found that when the 980 nm laser power density was below 3 MW/cm^2^, the generated optical force is not enough to confine the UCNP within the optical trap. For laser power densities ≥3 MW cm^–2^, the appearance of bubbles at the laser focus makes UCNP trapping impossible (see video S11 in the Supporting Information). Indeed, microbubbles can be generated near PNPs when the laser-induced temperature increase reaches Δ*T* ≈ 220 K.^37^ This local temperature increment is given by:^38^
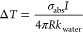
1where σ_abs_ is the absorption cross-section of PNPs, *I* is the laser power density, *R* is the radius of Au PNP, and *k* is the thermal conductivity of the surrounding medium (water). The calculated scattering, absorption, and extinction spectra of the PNPs are given in section S3, Supporting Information. Figure 2d shows the calculated local temperature increase as a function of the 980 nm laser power density for the two PNPs used in this work. For the PNPs with λ_SPR_ ≅ 980 nm, the critical temperature increment leading to bubble formation is achieved when the 980 nm laser power reaches 3.3 MW cm^–2^, in good agreement with the experimental observations. Thus, calculations and experiments reveal that stable OT of a single UCNP in the proximity of PNPs is not possible when trapping radiation is strongly absorbed by PNPs. In other words, the formation of bubbles avoids exploring the effect of plasmon-enhanced luminescence in a single optically trapped UCNP. It is important to note that when using the 95 nm Au PNPs, the absorption of 980 nm radiation is reduced but not eliminated. Consequently, thermal gradients are created in the surroundings of the optical tweezers, so that the thermophoretic effects cannot be disregarded. Under our experimental conditions, we have found that these effects increase the force acting on the UCNP (see section S4, Supporting Information).

Figure 2d reveals that when the OT wavelength is not resonant with λ_SPR_, OT laser power densities over 7.5 MW cm^–2^ (well above that required for stable tweezing of a single UCNP) can be used without leading to bubble formation. Indeed, stable OT of a single UCNP in the proximity of PNPs with λ_SPR_ ≅ 574 nm is possible in a wide range of 980 nm laser power densities (1.8–9.4 MW cm^–2^). The real-time fluorescence image of the OT allows us not only to evidence the OT of a single UCNP (bright spot in Figure 2h) but also to monitor the luminescence intensity when the UCNP is scanned in the surroundings of PNPs. To demonstrate the plasmon-enhanced luminescence, we first scanned a single UCNP parallel to the substrate, passing through a region of bare glass (without PNPs) and through a region containing the PNPs (see Figure 3a and Supporting Information, Figure S1). The OT–substrate distance, set by the vertical position of the laser focus, was kept at 100 nm. The analysis of UCNP luminescence intensity reveals a plasmon-enhancement close to 20% (Figure 3c and section S5.1, Supporting Information). Note that, while the upconversion intensity is quite homogeneous when the OT is scanned over the bare glass substrate, it becomes highly inhomogeneous when the OT is scanned on top of the PNPs. This can be explained by the inhomogeneity of PNPs deposited on the glass substrate and by considering that plasmon-enhancement depends on not only the vertical OT-PNP distance but also the in-plane OT-PNP relative position. To further demonstrate the plasmon-enhanced upconversion, we ran a second round of experiments, in which the OT was placed on top of PNPs. The luminescence intensity was registered as a function of the OT–substrate vertical distance (Figure 3b). The upconversion intensity was compared to that obtained when the same experiments were performed on top of a bare glass substrate, where no plasmon enhancement is expected (Figure 3d). While, in the absence of PNPs, the intensity remains independent of the OT–substrate vertical distance, a remarkable dependence on the OT–substrate distance is observed in the presence of the PNPs. In agreement with the experimental results of Figure 3c, the upconversion enhancement is close to 20% larger at the shortest OT–substrate vertical distance. Longer OT–substrate distances lead to reduced plasmon-induced enhancements. For OT–substrate distances above 1500 nm, the upconversion intensity is close to the values measured on the bare glass region; i.e., for this distance, there is no plasmon-enhanced upconversion luminescence.

**Figure 3:**
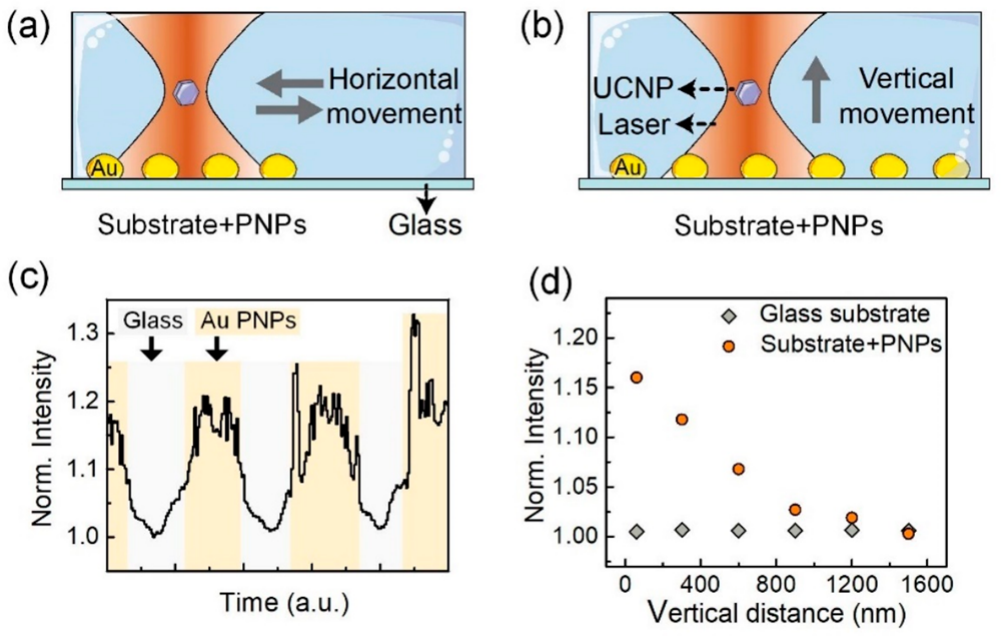
(a) Schematic representation of the horizontal scan of an optically tweezed UCNP along a substrate partially covered with Au PNPs. (b) Schematic representation of the vertical scan of an optically tweezed UCNP with respect to the substrate with PNPs. (c) Periodic change in the upconversion luminescence intensity during the horizontal scan of a single UCNP along a substrate partially covered with PNPs. (d) Upconversion intensity generated by a tweezed UCNP during a vertical scan. Data obtained in the presence and absence of PNPs are included for comparison. All of the data included in this figure were obtained for a laser power intensity of 4.3 MW cm^–2^ and for the PNPs with λ_SPR_ ≅ 574 nm.

There are two significant differences between our results and those reported for static UCNP-PNPs systems. First, the plasmon-induced enhancement here is moderate (20%) compared to those reported under “static” conditions (up to several orders of magnitude). Second, our results arise from a long-range effect, extending up to UCNP-PNP separations as large as 1 μm. On the contrary, the plasmon-induced luminescence enhancement had a short-range character (tens of nanometers at maximum) under static conditions. These discrepancies and the mechanisms behind them are discussed next.

The upconversion enhancement observed experimentally may be induced by the Purcell effect (an increase in the local density of photonic states at the emission wavelength) or by the local enhancement of the excitation field. We investigate the possibility of Purcell effect first because the emission wavelength of the UCNPs overlaps the plasmon resonance band of the Au PNPs (λ_SPR_≅574 nm). The position of the UCNP is parametrized in the calculations through its vertical distance to the substrate on which PNPs are deposited (*z*_UCNP_ in Figure 4a) and the UCNP-PNP in-plane distance (Δ*x* distance in Figure 4a). Figure 4b shows that, at vertical distances above half-wavelength (490 nm), the Purcell factor is 1 so the UCNP emission is not affected by the Purcell effect. However, in the near field of the PNPs (*z*_UCNP_ < 200 nm), the Purcell factor deviates from unity, becoming strongly dependent on Δ*x* (Figure 4b). Indeed, when the UCNP is optically trapped on top of a PNP (Δ*x* = 0), the Purcell effect decreases the emitted intensity (quenching), whereas when at Δ*x* = 285 nm it yields a ca. 30% increase in the upconversion luminescence (Figure 4b). This dependence on Δ*x* is particularly important in our case. An optically tweezed colloidal UCNP fluctuates continuously within the trap under Brownian motion. The dynamics of the UCNP in the horizontal plane are determined by the radial trap stiffness (*k*_*x*_). As discussed above, the trap stiffness was experimentally estimated by using the hydrodynamic drag method (see Figure 4c and section S5.2 in the Supporting Information). At the laser power used in the experiments of Figure 3 (23 mW, corresponding to a power density of 4.3 MW·cm^–2^), the force acting on a UCNP is 0.04 pN, corresponding to a *k*_*x*_ value of 78 nN/m. According to numerical simulations, the UCNP is weakly confined within the trap for that stiffness (Figure 4d, the simulation was conducted by using the Brownian Disk Lab,^39^ see details in section S6, Supporting Information). Indeed, the lateral distance between the UCNP and the longitudinal axis of the trapping beam fluctuates within a Δ*x* ≈ ±0.5 μm range. Under these conditions, the Purcell factor should be averaged within this broad range of in-plane UCNP-PNP relative positions. With such averaging, the enhancement in upconversion intensity caused by the Purcell effect is almost negligible (Figure 4e). Even for deeply subwavelength vertical distances, *z*_UCNP_ < 100 nm, the calculations reveal an almost negligible Purcell factor (≅ 1.05, Figure 4e). Thus, numerical calculations suggest that the Purcell effect is not the origin of the plasmon-induced enhancement of upconversion luminescence. This conclusion is confirmed by experimental measurements of the fluorescence lifetime. As the Purcell effect affects the spontaneous emission rate, it should induce a shortened luminescence lifetime of the UCNPs.^23,24,29,40−43^ We compared the luminescence decay curves (λ_exc_ = 980 nm and λ_em_ = 650 nm) of the UCNPs in the absence/presence of PNPs (Figure 4f, see measurement method in section S5.3, Supporting Information). The luminescence lifetimes are 443 and 430 μs, respectively. This difference is within the experimental uncertainty (±5% ≈ ±20 μs), and therefore the PNPs cause a negligible change in the radiative decay rate of the UCNPs.

**Figure 4:**
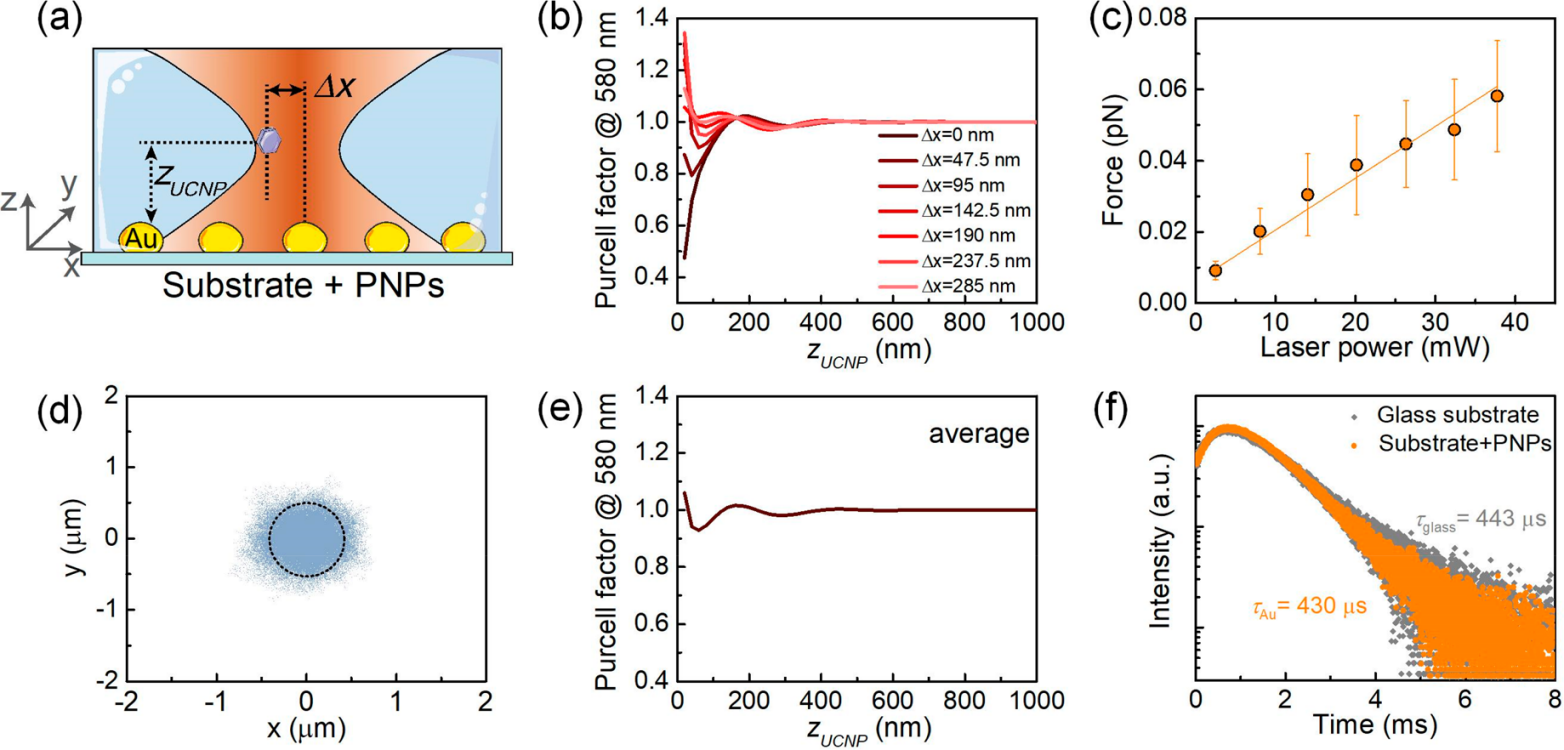
(a) Sketch of the position of the UCNP within the optical trap. Δ*x* sets the horizontal position of the laser focus, referenced to the position on top of the Au PNP. (b) Dependence of the Purcell factor at 580 nm on the vertical direction. Different colors correspond to the vertical scans performed at different positions (different Δ*x* distances) with respect to the Au PNP. (c) Experimentally determined, laser-power-dependent force for optical trapping of a single UCNP on a substrate with PNPs. Solid lines plot linear fits to the experimental data. (d) Brownian motion trajectory of a single optically trapped UCNP within the transverse section of the laser focus (indicated by the black dashed circle). (e) Dependence of the in-plane spatially averaged Purcell effect at 580 nm along the vertical direction. (f) Upconversion luminescence decay curves recorded for an emission wavelength of 650 nm for UCNPs deposited on a glass substrate (gray) and on a glass substrate containing PNPs (orange) under 980 nm excitation. All of the data included were obtained with the PNPs with plasmon resonance at 574 nm.

Once the Purcell effect is discarded as the mechanism behind the upconversion luminescence enhancement, we evaluate the increase in absorption efficiency induced by the plasmon-enhanced local-field confinement of 980 nm radiation. Numerical simulations provide the electric field distribution of the 980 nm laser beam along the vertical direction, *z*_UCNP_, for different vertical positions of the laser focus, *z*_L_, both measured from the substrate surface (see sections S3, S7, and S8, Supporting Information). We consider *z*_L_ to be between 0 and 2000 nm above the substrate. For each focus position, the 980 nm laser intensity is estimated along the *z* direction, normalizing it to the free-space intensity at each point (*I*_980_^*n*^). Numerical calculations reveal that the 980 nm intensity is strongly (6-fold) enhanced at positions in proximity (*z*_UCNP_ < 80 nm) to the substrate (Figure 5a). Remarkably, the laser intensity profile is almost independent of the focus position, as it is fully governed by the plasmonic tail in the vicinity of the PNPs and the oscillations induced by the substrate reflection (note that all of the curves in Figure 5a overlap almost exactly). In our experimental conditions (see Section S9, Supporting Information), the upconversion emission increases linearly with the laser intensity. Thus, the 6-fold enhancement in the excitation intensity predicted numerically (Figure 5a) would lead, at short UCNP–PNP distances, to a 6-fold luminescence enhancement. However, the numerical and experimental data differ both in magnitude and spatial range of luminescence enhancement. The experimental data show a moderate 20% enhancement which extends for hundreds of nanometers in the vertical direction, whereas theoretical calculations predict a 6-fold enhancement that is produced only in very close proximity to PNPs. Note that this comparison between theory and measurements is based on a crude assumption: numerical calculations assume that the UCNP is always placed at the laser focus (this is the only vertical distance we have access to experimentally). In other words, the calculations in Figure 5a assume a deterministic UCNP–PNP distance that is far from the real situation: Brownian motion makes the UCNP vertical position fluctuate continuously. Indeed, simulations indicate that the Brownian-induced fluctuation in the vertical distance can be larger than Δ*z*_UCNP_ ≈ ±1 μm (Figure 5b and section S6 in the Supporting Information).

**Figure 5:**
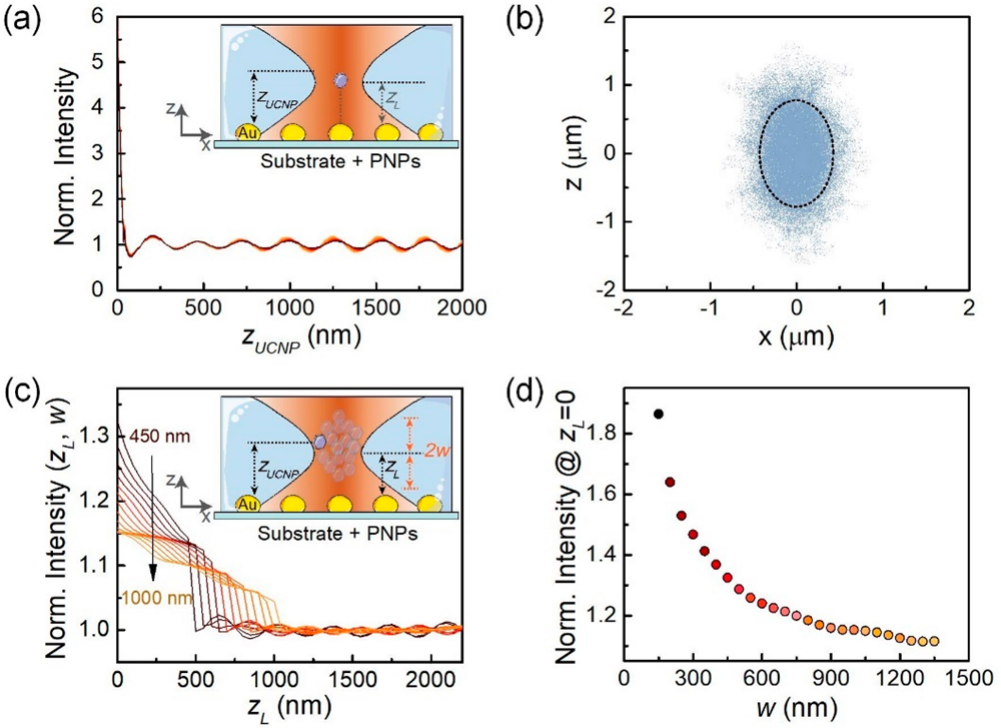
(a) Spatial profile of the laser intensity on top of a PNP and along the vertical distance (*z*, measured from the substrate on which PNPs are deposited) as obtained for different values of *z*_UCNP_. The profiles for laser position, *z*_L_ ranging between 0 and 2000 nm, overlap almost completely. Inset: schematic of the UCNP on top of a Au PNP. (b) Position distribution of a single optically tweezed UCNP caused by Brownian motion within a longitudinal section of the laser beam axis. The black dashed ellipse indicates the Rayleigh range (long axis) and the size of the laser focus (short axis). (c) Dependence of the effective intensity (experienced by the Brownian UCNP) as a function of the OT vertical position, *z*_L_, and for half position distribution widths, *w*, ranging from 450 nm (black) to 1000 nm (orange). Inset: sketch of the UCNP at the on-top position of the Au nanoparticle. (d) Effective 980 nm laser intensity for *z*_L_ = 0 nm as a function of the width of the UCNP position distribution.

To take the vertical Brownian motion of the UCNP into account, we refine the theoretical model (see details in Section S10, Supporting Information). We describe the vertical position of the UCNP through a uniform distribution centered at the laser focus, which enables us to explore the intensity profiles in Figure 5a around *z*_L_. As shown in the inset of Figure 5c, the half-width of the UCNP position distribution, *w*, is set to values between 450 and 1000 nm (in accordance with Figure 5b). For *z*_L_ < *w*, the lower bound in the distribution width was set by the substrate (the UCNPs cannot trespass it). For each laser focus position, *z*_L_, we convolute the calculated 980 nm laser intensity profiles in Figure 5a with the UCNP position distributions for different *w*. Hence, the laser intensity is estimated by averaging the position distribution experienced by the colloidal (moving) UCNP, *I*_980_^*n*^. This effective intensity is now a function of both *z*_L_ and *w*. Figure 5c shows *I*_980_^*n*^(*z*_L_) curves for *w* ranging from 450 nm (black) to 1000 nm (orange). The maximum effective intensity enhancement is obtained at *z*_L_ = 0, *I*_980_^*n*^ (*z*_L_ = 0), whose value decreases as the width of the UCNP distribution increases (Figure 5d). This simply reflects that the plasmon-induced upconversion enhancement experienced by the UCNP becomes larger as its fluctuation near the PNPs decreases. Although the OT is placed very close to the PNP, the actual UCNP–PNP distance fluctuates, so that the UCNP spends a large fraction of time at positions where the plasmon contribution to the laser intensity is negligible. Indeed, our numerical model reveals that the Brownian position distribution width must be set to 900 nm to reproduce the experimentally obtained plasmon-induced enhancement (ca. 1.17). This is in very good agreement with the position distribution of UCNP within the trap included in Figure 5b.

In summary, we investigated the plasmon-enhanced luminescence in a single colloidal upconverting nanoparticle. By using single-beam optical tweezers, we achieved three-dimensional manipulation of an individual upconverting nanoparticle in the surroundings of plasmonic nanoparticles immobilized on a transparent substrate. Our findings reveal challenges in achieving stable trapping when the trapping radiation overlaps with the plasmon resonance due to bubble formation caused by local heating. When trapping radiation is weakly overlapping with the plasmonic band, three-dimensional scanning of a single upconverting nanoparticle on top of the plasmonic nanoparticles becomes possible. Under these conditions, experimental data revealed a 20% enhancement in the luminescence intensity for small tweezing distances (<1 μm). The comparison between experimental data and numerical simulations dismisses the Purcell effect as the primary cause of luminescence enhancement. Instead, we concluded that the enhancement is attributed to the plasmon-induced local-field confinement, leading to improved absorption efficiency. Numerical simulations and experimental data reveal differences in the magnitude and spatial range of luminescence enhancement under the assumption of deterministic optical tweezing. Once the impact of Brownian motion on UCNP position distribution is considered, and refined theoretical models are proposed for the fluctuation in distance between upconverting and plasmonic nanoparticles, a remarkable agreement between theory and experiments is obtained.

This work provides valuable insights into the challenges and opportunities of using plasmonic substrates to enhance the brightness of colloidal upconverting nanoparticles. The findings contribute to the understanding of the complex interplay among plasmonic effects, optical trapping, and Brownian motion in colloidal systems, paving the way for future advancements in the field of photonics and nanotechnology.

## Supporting Information Available

Synthesis of the UCNPs, fabrication of the plasmonic substrate, experimental details, simulation of Brownian motion, numerical simulation models, and methods (PDF)
Bubble formation due to excessive heating (video)

## Abbreviations

UCNPs: upconverting nanoparticles
PNSs: plasmonic nanostructures
OTs: optical tweezers
PNPs: plasmonic nanoparticles
TEM: transmission electron microscope
CCD: charge-coupled device
SPR: surface plasmon resonance
