# Supplementary material for: Brownian Motion Governs the Plasmonic Enhancement of Colloidal Upconverting Nanoparticles
Source: Nano Lett. 2024 Mar 18;24(12):3785–92. doi: 10.1021/acs.nanolett.4c00379 (PMC10979430; doi:10.1021/acs.nanolett.4c00379)
Supplement: Supplementary file 1 — nl4c00379_si_001.pdf [file nl4c00379_si_001.pdf]

Supporting Information

Brownian motion governs the plasmonic enhancement of colloidal upconverting nanoparticles

Fengchan Zhang[1,2], Pedro Ramon Almeida Oiticica[3], Jaime Abad-Arredondo[4], Marylyn Setsuko Arai[3], Osvaldo N. Oliveira Jr. [3], Daniel Jaque[1,5,*], Antonio I. Fernandez Dominguez[4,*], Andrea Simone Stucchi de Camargo[6,7,*], and Patricia Haro-González[1,2,5,*]

[1]Nanomaterials for Bioimaging Group (nanoBIG), Departamento de Física de Materiales, Facultad de Ciencias, Universidad Autónoma de Madrid, Madrid, 28049, Spain

[2]Instituto Nicolás Cabrera, Facultad de Ciencias, Universidad Autónoma de Madrid, Madrid, 28049, Spain

[3]São Carlos Institute of Physics, University of São Paulo (USP), 13566-590 São Carlos, SP, Brazil

[4]Departamento de Física Teórica de la Materia Condensada and Condensed Matter Physics Center (IFIMAC), Facultad de Ciencias, Universidad Autónoma de Madrid, E28049 Madrid, Spain

[5]Institute for Advanced Research in Chemical Sciences, Facultad de Ciencias, Universidad Autónoma de Madrid, 28049 Madrid, Spain

[6]Federal Institute for Materials Research and Testing (BAM), 12489, Berlin, Germany

[7]Friedrich Schiller University (FSU), Jena, 07737, Germany

*Corresponding authors

Table of Content

S1. SYNTHESIS AND CHARACTERIZATION OF NAYF$_4$: 25%YB^{3+}, 0.3%TM^{3+}@NAYF$_4$ 3

S2. FABRICATION AND CHARACTERIZATION OF PLASMONIC SUBSTRATES 4

S3. CALCULATED SCATTERING, ABSORPTION, EXTINCTION, AND PURCELL SPECTRA OF AU PNPS 6

S4. OPTICAL TRAPPING OF UCNP UNDER THERMOPHORETIC EFFECTS 8

S5. EXPERIMENTAL DETAILS 10

S5.1 MEASUREMENT OF THE LUMINESCENCE INTENSITY 10
S5.2 MEASUREMENT OF FORCES FOR OPTICAL TRAPPING OF A UCNP 10
S5.3 MEASUREMENT OF FLUORESCENCE LIFETIME 10

S6. SIMULATION OF BROWNIAN MOTION 11

S7. NUMERICAL SIMULATIONS OF THE ELECTRIC FIELD 13

S8. SIMULATION DOMAIN AND FIELD PROFILES 14

S9. DEPENDENCE OF UCNP EMISSION INTENSITY ON LASER POWER 16

S10. EFFECTIVE BEAM INTENSITY AND BROWNIAN MOTION 17

S11. VIDEO: BUBBLE FORMATION DUE TO EXCESSIVE HEATING. 18

S1. Synthesis and characterization of $NaYF_4$: 25%Yb^{3+}, 0.3%Tm^{3+}@$NaYF_4$

The upconverting nanoparticles (UCNP) were synthesized following a high-temperature co-precipitation method previously reported by Gnanasammandhan et al.[1] To prepare the core $NaYF_4$: Yb^{3+}, Tm^{3+} nanocrystal, $LnCl_3$ aqueous solutions (0.747 mL YCl_3 (1M), 0.25 mL $YbCl_3$ (1M) and 0.30 mL $TmCl_3$ (0.01M)), were transferred to a 100 mL three-necked round-bottom flask and heated until complete water evaporation. Subsequently, the resulting powder was mixed with 6 mL oleic acid and 15 mL octadecene, heated to 150 °C for 30 min under an argon atmosphere to form a homogeneous solution, and then cooled down to room temperature. 5 mL of methanol solution containing NaOH (0.1 M) and NH_4F (0.148 g) were slowly added into the flask and quickly formed solid-state precipitates in the solution. Subsequently, the solution was slowly heated to 110 °C to evaporate methanol, degassed for 10 min, and then heated to 300 °C and maintained for 1h under an inert atmosphere. After the solution was naturally cooled down, nanocrystals were precipitated with acetone, isolated by centrifugation (6000 rpm, 10 min), and washed once with acetone and twice with ethanol. In a subsequent step, the core UCNPs were coated with an undoped matrix shell, following a similar synthesis procedure. 1 mL of YCl_3 aqueous solution (1M) was transferred to a 100 mL three-necked round-bottom flask and heated until dryness. Then, the resulting powder was mixed with 6 mL OA, and 15 mL ODE, and heated to 150 °C for 30 min to form a yellow homogeneous and clear solution. After cooling to room temperature, as-prepared UCNPs (re-dispersed in 15 mL of cyclohexane) were added to the above solution and the mixture was heated to 100 °C. After removing cyclohexane, the synthesis proceeded following the same steps as that of the core $NaYF_4$:Yb^{3+}, Tm^{3+} nanoparticles. The final core-shell nanocrystals were washed with acetone one time, with ethanol two times, and dried at room temperature.

The produced UCNPs exhibit a hexagonal structure and a size of approximately 43 nm. Their upconversion emission spectrum upon excitation at 980 nm presents emissions at 347 and 362 nm assigned to the $^1I_6 \rightarrow {}^3F_4$, and $^1D_2 \rightarrow {}^3H_6$ transitions, and at 452, 476, 649 and 700 nm corresponding to the $^1D_2 \rightarrow {}^3F_4$, $^1G_4 \rightarrow {}^3H_6$, $^1G_4 \rightarrow {}^3F_4$ and $^1G_4 \rightarrow {}^3H_5$ transitions.

S2. Fabrication and characterization of plasmonic substrates

The plasmonic substrates consists of Au plasmonic nanoparticles (PNPs) attached to the glass surface. They were fabricated by Au ultrathin film evaporation onto bare glass substrates, followed by thermal annealing.[2, 3] The morphology and localized surface plasmon resonance (LSPR) spectral response of the plasmonic substrates were controlled by the fabrication parameters. The fabrication procedure was as follows. The glass slides with dimensions 25 × 8 × 1.0 mm^3 were cleaned using ultrasonic thermal bath at 60 °C in neutral detergent solution for 20 min, ultrapure Milli-Q water for 10 min, and isopropanol for 10 min. Then the substrates were treated using UV/Ozone for 20 min, rinsed in ultrapure water, and dried under a flow of nitrogen. An ultrathin gold film (Au/glass) is formed by thermal evaporation using the MB-Evap inside a LabMaster 130 Glovebox (MBraun) at a chamber pressure 1×10^{-6} mBar and film growth rate 0.03 nm/s. The growth rate and gold film thickness were automatically controlled during deposition using a quartz crystal microbalance inside the evaporation chamber. The Au/glass films were annealed inside a furnace muffle. During the thermal annealing, the Au PNPs are formed on the glass surface by coalescence and are partially embedded to the glass substrate increasing the Au-glass adherence. Finally, the plasmonic substrates were cleaned with ultrasonic thermal bath with isopropanol for 10 min. To obtain plasmonic substrates composed of Au PNPs with different diameters and therefore different LSPR bands (λ_{SPR}), used in this work we used different Au film thickness, annealing time, and temperature. The set of parameters are shown in **Table S1**. A bare glass region was produced in the same glass slide (**Figures S1a** and **S1b**), by adding a mask onto the glass surface before the Au film evaporation step and kept the other fabrication steps.

Table S1. Fabrication parameters of plasmonic substrates with different Au PNPs plasmonic resonances.

Plasmonic Substrate	Au thickness (nm)	Annealing Temperature (°C)	Annealing Time (h)
$\lambda_{SPR} \cong 574\ nm$	6	600	4
$\lambda_{SPR} \cong 980\ nm$	25	600	6
$\lambda_{SPR} \cong 548\ nm$ (half-deposited)	6	610	2

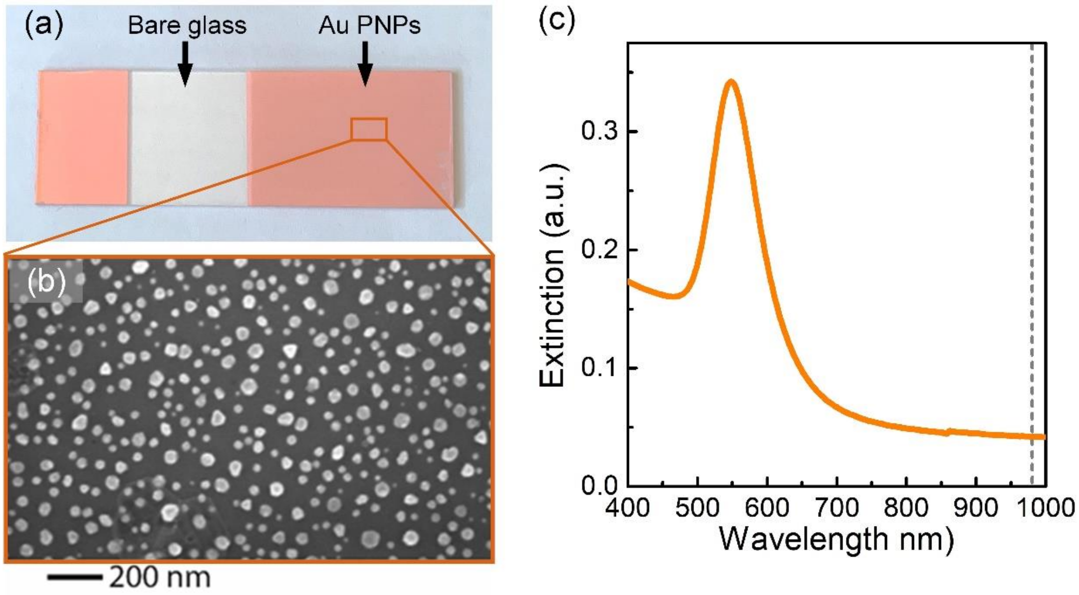

Figure S1. (a) The substrate partially covered with Au PNPs. **(b)** Scanning electron microscope image of the Au PNPs. **(c)** LSPR spectrum of this plasmonic substrate with maximum plasmon resonances at approximately $\lambda_{SPR} \cong 548$ nm

---

The LSPR spectrum of the plasmonic substrate is determined by the dimension of the Au PNPs and the interparticle distance distributions. The substrates covered with larger Au PNPs have maximum resonance at longer wavelengths. The distribution of nanostructure sizes influences the FWHM of the LSPR spectrum and the interparticle distances has influences on the spectrum shape due to coupled plasmonic oscillations.[4, 5] **Figure S1c** shows the LSPR spectrum of the Au PNPs. **Table S2** shows the particle analysis results from the scanning electron microscope images of the plasmonic substrates used in this work.

Table S2. Particle analysis results from scanning electron microscope images for Au PNPs diameter, interparticle distances (between particle edges), particle density and percent of surface coverage.

**Plasmonic Substrate**	**Particle Diameter (nm)**	**Interparticle Distance (nm)**	**Particle Density (μm^{-1})**	**Surface Coverage (%)**
$\lambda_{SPR} \cong 574\ nm$	95 ± 6	79 ± 43	18.6 ± 0.9	9.6 ± 0.3
$\lambda_{SPR} \cong 980\ nm$	264 ± 40	178 ± 67	4.1 ± 0.2	18.5 ± 0.5
$\lambda_{SPR} \cong 548\ nm$ (half deposited)	31 ± 11	24 ± 10	185 ± 13	15.7 ± 1.0

S3. Calculated scattering, absorption, extinction, and Purcell spectra of Au PNPs

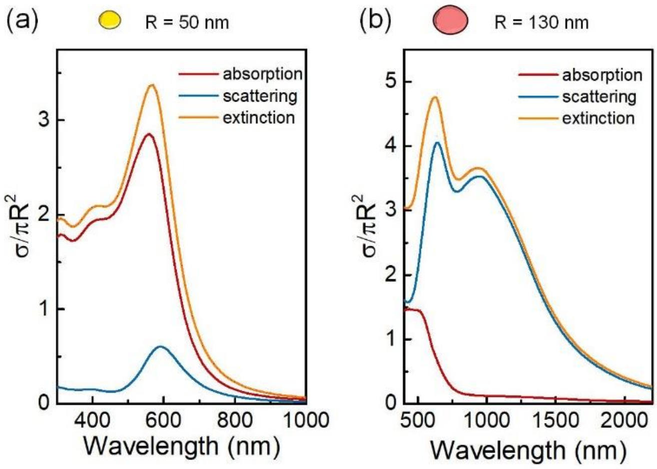

Figure S2. Calculated absorption, scattering and extinction cross-section spectra (normalized to the geometrical cross section) for Au PNP with radius of **(a)** 50 nm **(b)** 130 nm.

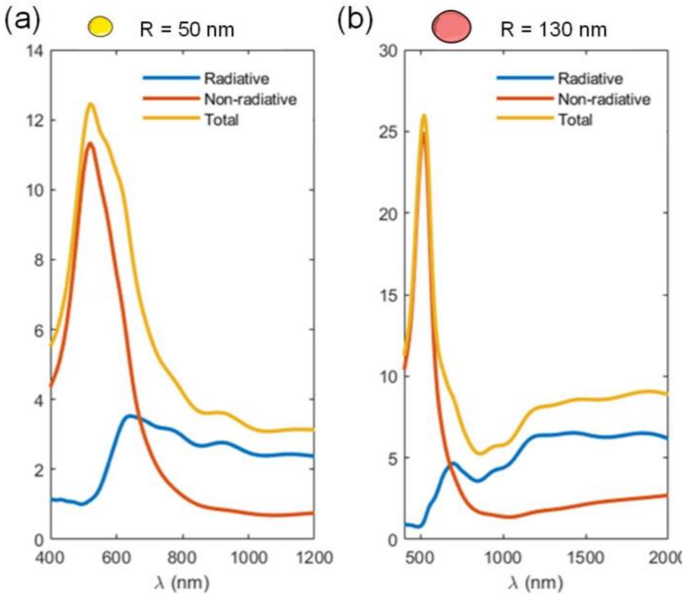

Figure S3. Calculated absorption, scattering and extinction cross-section spectra (normalized to the geometrical cross section) for an Au PNP with radius of **(a)** 50 nm **(b)** 130 nm.

Although there is an experimental challenge by using the PNPs with $\lambda_{SPR} \cong 980$ nm due to the excessive heat, we explore its potential contribution to the upconversion enhancement by numerical calculation. **Figure S2** shows the lager PNPs scatter light more efficiently than the absorption, which means a higher reflectivity of the effective medium they would form. This would translate into a larger contrast of the field oscillations along the vertical direction observed in **Figure 5a**, that could compromise the stability of the optical tweezers. Moreover, their lower absorption (compared with the scattering) means a lower field near-field localization. Therefore, the PNPs with $\lambda_{SPR} \cong 980$ nm would not effectively increase the magnitude of local field enhancement.

Figure S3 shows Purcell factor calculations evaluated 50 nm away from the PNP surface. At distances as small as the PNP radius itself, the Purcell factor has dropped to moderate values, ~4. The Purcell factors for both PNP sizes are very similar at the minimum distances probed in our experiment. Therefore, the Purcell effect plays a minimal role for larger PNPs as well.

S4. Optical trapping of UCNP under thermophoretic effects

Our simulations reveal that for the laser power density used in our experiments (4.3 MW/cm^2) the temperature increment caused by plasmonic nanoparticles at the laser focus could be as large as 40 K. The presence of a thermal gradient in the surroundings of the laser spot could strongly affect the dynamics of the optically trapped nanoparticle. As it is clearly explained by S. Liu et al. when a nanoparticle is within a thermal gradient, various kinds of thermophoresis-correlated effects can be activated, leading to the appearance of different forces that can be attractive/repulsive in respect to the thermal gradient.[6] In our case (nanoparticles suspended in a polar solvent as water) these effects can be restricted to the appearance of dispersion forces (pushing particles from the hot to the cold areas, i.e. in opposite direction to the thermal gradient) and of interfacial-entropy-driven forces. The latter ones are caused by the permittivity gradient induced at the surface of the nanoparticle due to the thermal gradient and results in a thermophoretic force that pushes the colloidal nanoparticle from the cold to the hot regions. This effect has been demonstrated to be strong enough to induce the trapping of biological cells by using laser-induced hot spots in a metallic substrate, as demonstrated by Y. Zheng et. al..[7] The interfacial-entropy-driven forces require the existence of a surface charge in the object/nanoparticle that is being manipulated, for instance the charge of the cell membrane (Zeta potential of +40 to -70 mV).

We have measured the charge of our upconverting nanoparticles: -11 mV (see **Figure S4**), so that we cannot discard the possible existence of interfacial-entropy-driven forces. Indeed, in our conditions (laser focus causing a local heating) we will have three different forces acting on our upconverting nanoparticles: the optical forces caused by the gradient in the laser electric field ($\overrightarrow{F_O}$, attractive forces), the dispersion forces ($\overrightarrow{F_D}$, , repulsive), and the interfacial-entropy-driven forces ($\overrightarrow{F_{ED}}$, attractive). The total force acting on the UCNP when it is located close to the laser focus ($\overrightarrow{F_T}$) is then given by:

$$\overrightarrow{F_T} = \overrightarrow{F_O} + \overrightarrow{F_D} + \overrightarrow{F_{ED}} \tag{S1}$$

It could happen that the repulsive forces dominate over the attractive forces and that it could be not possible to push the nanoparticle in proximity towards the substrate. To evaluate this possibility, we have measured $\overrightarrow{F_T}$ -its radial component- in presence and absence of the plasmonic nanoparticles (i.e. in presence and absence of local heating) by the hydrodynamic drag method. Results are included in **Figure S5**. Experiments reveal that the total force increase due to the presence of the plasmonic nanoparticles. The enhancement in the total force acting on the single UCNP cannot be only explained in terms of the existence of an attractive interfacial-entropy-driven force but also on a plasmon-induced enhancement in the optical forces (due to the local enhancement of 980 nm radiation). At this point, we are not in conditions to elucidate the origin of this improvement in $\overrightarrow{F_T}$ and further experiments will be necessary. But what we can claim is that the thermal related effects are not avoiding the positioning of the upconverting nanoparticle close to the plasmonic nanoparticles. Indeed, these preliminary data reveal that thermophoretic effects in our case are positive and tend to attract the upconverting nanoparticle towards the plasmonic nanostructures.

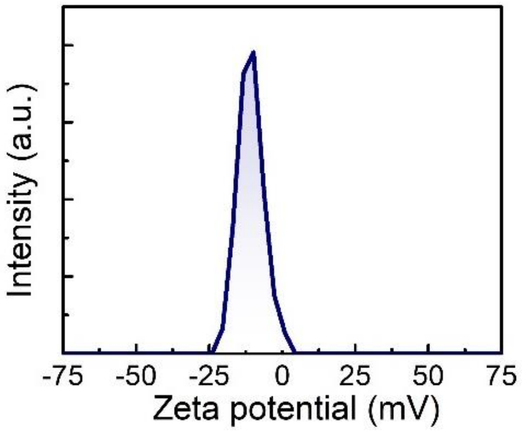

Figure S4. Zeta potential of the upconverting nanoparticles.

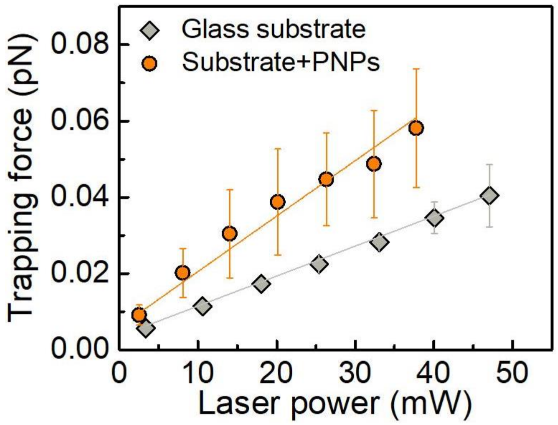

Figure S5. Experimentally determinate laser power dependent force for optical trapping of a single UCNP on a glass substrate (grey) and a substrate with PNPs, respectively.

S5. Experimental details

S5.1 Measurement of the luminescence intensity

The luminescence intensity generated by a single UCNP under the excitation and trapping of 980 nm laser was obtained by processing the fluorescence images taken with a CCD camera. The luminescence intensity was analyzed by using ImageJ software. The bright spot was selected by a circular region of interest (ROI), and the luminescence intensity of the whole region was obtained. The size and shape of the ROI for the intensity of the background was the same as that used for the bright spot. The actual luminescence intensity from the optically trapped UCNP was obtained by subtracting the background from the luminescence intensity of the whole region.

S5.2 Measurement of forces for optical trapping of a UCNP

The force can be determined experimentally by the hydrodynamic drag method. It consists of measuring the drag force F_{drag} determined by the fluid velocity v: $F_{drag} = 6\pi\eta r v$, where η is the viscosity of the fluid (water in this work), r is the hydrodynamic radius of the trapped object. This force will drag the particle away from its equilibrium position on the trap when it overcomes the total force for trapping. The total force was therefore calibrated from the escape velocity. The motorized translation stage in the experimental set-up allowed to induce a relative velocity between the optically trapped UCNP and the surrounding medium.

S5.3 Measurement of fluorescence lifetime

The suspension of UCNPs were dropped and dried on the bare glass and the substrate coated with Au PNPs, respectively. A 980 nm pulsed laser with a repetition frequency of 10 Hz, a duration time of 8 ns, and a pulse energy of 19 J was used to illuminate the UCNPs deposited on the substrates. The emission signal was collected by a set of lenses and filtered by a monochromator. The detection wavelength was set at 650 nm. The emission intensity decay was recorded by a visible photomultiplier connected to an oscilloscope. The data was obtained by an average of 3000 scans.

S6. Simulation of Brownian motion

The Brownian motion trajectories of the UCNP within the optical trap were simulated by using Brownian Disk Lab (BDL) software.[8] The size of UCNP, size of optical trap, temperature, viscosity and trap stiffness were included as a simulation parameter applied to particle. The temperature was set to 298 K. The horizontal trap stiffness was experimentally determined by the hydrodynamic drag method. At the laser power used in the experiments of Figure 3 (23 mW), we estimate a force of 0.04 pN, corresponding to a horizontal trap stiffness k_x of 78 nN/m.

The trap stiffness along the laser axial direction, k_z, was theoretically calculated.

For the nanoparticle with size much smaller than the wavelength of trapping laser ($R \ll \lambda$), it can be considered as a dipole. It can be considered that the gradient force dominates the confinement of the nanoparticle. It is given by:

$$F_{grad} = \frac{1}{4}\alpha_{NP}\nabla|E|^2 \tag{S2}$$

where $|E|^2$ is the time averaged square of the electromagnetic field, α_{NP} is the polarizability of the nanoparticle. Because the intensity of the electromagnetic is $I = \frac{1}{2}\frac{\alpha_{NP}}{c\varepsilon_0}|E|^2$, the gradient force can be written in terms of the gradient of intensity as

$$F_{grad} = \frac{1}{2}\frac{\alpha_{NP}}{c\varepsilon_0}\nabla I \tag{S3}$$

where c is the speed of light, ε_0 is the dielectric constant of vacuum.

To approximately analyze the gradient intensity experienced by the nanoparticle, we can consider the intensity distribution the trapping beam to be Gaussian:

$$I(r,z) = I_0\left(\frac{\omega_0}{\omega(z)}\right)^2 exp\left(\frac{-2r^2}{\omega(z)^2}\right) \tag{S4}$$

where I_0 is the maximum intensity, r is radial coordinate in the transverse plane, ω_0 is the beam waist, and $\omega(z) = \omega_0\sqrt{1+\frac{z^2}{z_R{}^2}}$ is the beam width along the axial direction, z. z_R is the Rayleigh range, which can be written as:

$$z_R = \frac{\pi\omega_0{}^2 n_{medium}}{\lambda} \tag{S5}$$

In the focus plane of the trapping beam, $z = 0$, $\omega(z = 0) = \omega_0$, for $r = \omega_0$, the intensity gradient is:

$$I(r) = I_0 e^{-2} \tag{S6}$$

$$\nabla I_r = -4\frac{I_0}{{\omega_0}^2} e^{-2} \propto F_{grad}(x). \tag{S7}$$

In the axial direction, $r = 0$, the intensity gradient is

$$I(z) = I_0 \left(1 + \frac{z^2}{{z_R}^2}\right)^{-1} \tag{S8}$$

$$\nabla I_z = -2\frac{I_0\, z}{{z_R}^2}\left(1 + \frac{z^2}{{z_R}^2}\right)^{-2}. \tag{S9}$$

For $z = z_R$,

$$\nabla I_z = -\frac{I_0}{2z_R} \propto F_{grad}(z). \tag{S10}$$

The optical trap in our experiment is generated by a focused 980 nm laser beam by using an oil immersion objective ($100 \times /NA$ 1.4). The radius of laser focus ω_0 is 427 nm, the Rayleigh range z_0 in water is 779 nm. The optical trapping forces can be written as

$$F_{opt(x)} = -k_x\omega_0 \propto -1.27\, I_0 \tag{S11}$$

$$F_{opt(z)} = -k_z\omega_z \propto -0.64\, I_0. \tag{S12}$$

Therefore, the axial trapping stiffness k_z used for the simulation of Brownian motion is estimated to be 22 nN/m.

S7. Numerical simulations of the electric field

To describe the electric field of the optical tweezer we employ COMSOL Multiphysics and use a scattered field formulation, in which the Gaussian beam acts as a background field. The beam profile is described analytically within the paraxial approximation. For an electric field polarized along $\hat{x}$, and that propagates in the $\hat{z}$ direction, with its focus at the origin, the electric field of the beam can be described through:

$$E_x = \mathcal{E} \cdot \sqrt{\frac{w_0}{w(z)}} \exp\left(-\left(\frac{x}{w(z)}\right)^2\right) \exp\big(i\eta(z) - ikz\, f(x,z)\big), \tag{S13}$$

with $f(x,z) = 1 + \frac{1}{2}\left(\frac{x}{z_R} \cdot \frac{w_0}{w(z)}\right)^2$, $w(z) = w_0\sqrt{1 + \left(\frac{z}{z_R}\right)^2}$, $\eta(z) = \frac{1}{2}\text{atan}\left(\frac{z}{z_R}\right)$, and $z_R = \pi w_0^2/\lambda$.

In **Equation 2**, w_0 is the waist of the beam at the focal point, λ and k are respectively the wavelength and wavevector of the incident field in the focusing medium, and $\mathcal{E}$ denotes the peak electric field amplitude at the focus point. Note that the beam is assumed to be homogeneous in the $\hat{z}$ direction. If the Gaussian beam carries a total power P, then the peak amplitude is given by

$$\mathcal{E} = i\left(\frac{2}{\pi}\right)^{\frac{1}{4}} \sqrt{\frac{Z_0}{n} 2P\, k \left(\frac{z_R}{w_0}\right) \frac{1}{kz_R\left(\left(\frac{w_0}{2z_R}\right)^2 + 2\right) - 1}}, \tag{S14}$$

with Z_0 being the vacuum impedance, and n is the refractive index of the focusing medium. Our simulation domain is a square prism with side of 2 μm and 3 μm height. Gold nanoparticles with permittivity given by Rakić et al.[9] are randomly scattered over a glass substrate (n=1.5), mimicking experimental samples. The simulation domain is terminated by scattering boundary conditions.

S8. Simulation domain and field profiles

Figure S6 shows sketches of the simulation domain considered in the simulation volume, 2 μm × 2 μm × 3 μm, for a small ensemble (50) of Au nanoparticles. The glass substrate is shown in red, and the Au PNPs in blue. These are distributed randomly, mimicking the experimental scanning electron microscope image in the right panel. We tested that their replacement with Floquet lateral boundary conditions did not alter the results, which means that diffractive (long-range, cooperative) effects were not playing a relevant role in the system.

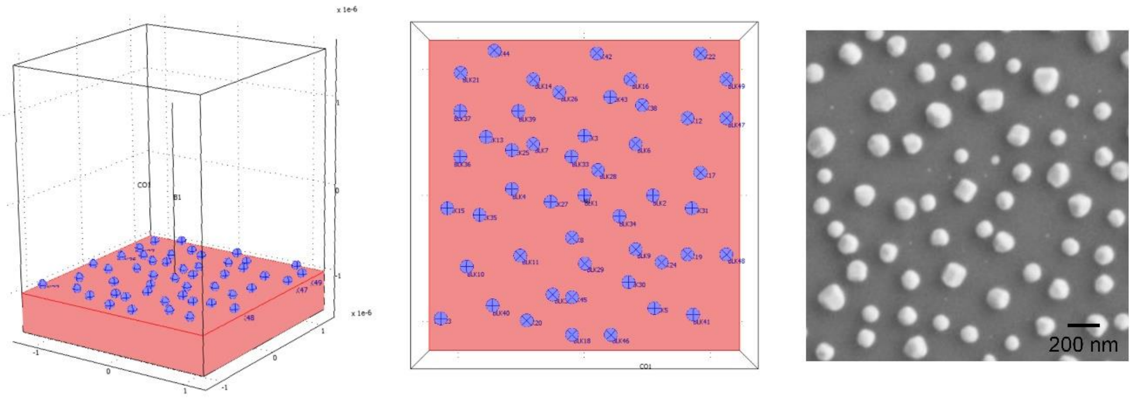

Figure S6. Sketch of the simulation volume employed in the calculations and its comparison against the experimental scanning electron microscope image (right).

Top panels in **Figure S7** show EM calculations for the gaussian beam propagation in three different media: free space (left), air-glass interface (center) and air-glass interface covered with Au PNPs (right). In all cases, the beam focus is located at the same position, 100 nm over the interface. In the left panel, the beam propagates without significant deviations from the analytical, paraxial field defined as the background input in COMSOL Multiphysics. In the central panel, maxima and minima emerging from the standing wave structure generated as a result of the reflection at the glass surface are apparent. In the right panel, this structure persists, but now, strong field enhancements (here with color saturation for clarity) take place in the near field of the PNPs. The color codes the electric field intensity in linear scale from black (minimum) to white (maximum) in a thermal-like scale.

The intermediate and bottom panels display the horizontal and vertical components, respectively, of the intensity gradient for to the three system configurations considered in the top panels. The optical force exerted by the beam on the UCNPs is proportional to this intensity gradient, which therefore indicates its sign and direction. In the intermediate panels, we observe that the beam traps the UCNPs horizontally along its longitudinal axis. This lateral confinement becomes stronger at the glass-air interface when the PNPs are placed on top, although this

effect is not clearly seen due to the color saturation introduced for clarity. In the bottom panels, we can observe that the beam pushes the UCNP towards the glass surface when it is placed along its longitudinal axis. Any lateral displacement away from the beam axis would induce a vertical force pushing the UCNP away along the positive, vertical direction. Again, the presence of the PNPs increases the magnitude of these forces in the near-field of the air-glass interface.

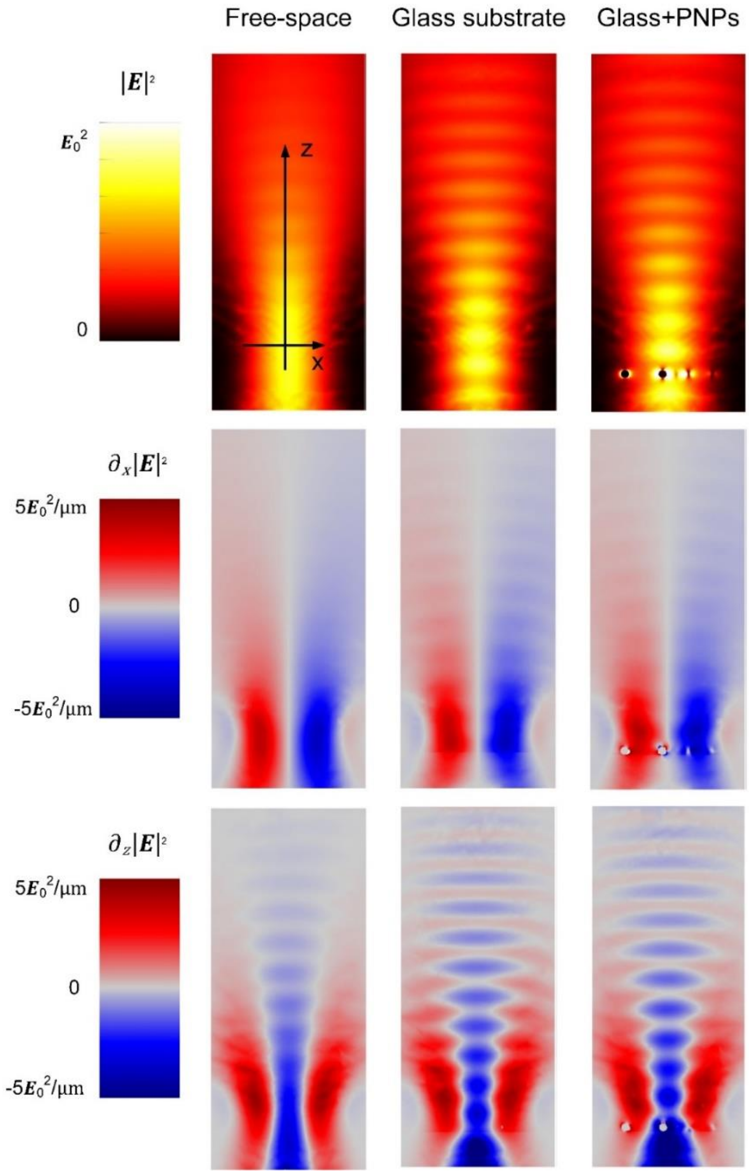

Figure S7. Calculated beam intensity profile (top) and its horizontal (intermediate) and vertical gradients (bottom) for three different structures: Free space (left), bare air-glass interface (center) and glass surface covered with PNPs (right).

S9. Dependence of UCNP emission intensity on laser power

At small laser focus-substrate distance, the normalized intensity is in a range of 1.15 -1.3 and drops to 1 when the distance is increased (w = 400 nm, it drops to 1 at z_L of 500 nm, w = 1000 nm, it drops to 1 at z_L of 1050 nm). The average intensity at w from 450 to 1000 nm is in good agreement with the experimental data shown in **Figure 3d**. These results lead us to conclude that the overall increase in luminescence intensity may attributed to the incident field intensity enhancement. In general, the emission intensity is proportional to E^{2n} for an n-photon upconversion process under moderate laser power irradiation. For the UCNP used in this work, a quadratic correlation remains between laser power density and emission intensity (650 nm) under the power density of 37 to 235 kW cm^{-2} (**Figure S8**). However, the laser power density we used for measuring the luminescence enhancement is up to 4.3 MW cm^{-2}. As the power density increases, the slope of the upconversion luminescence changes from quadratic to linear due to the competitive mechanisms of upconversion and downconversion for the depletion of the intermediate excited state.[10, 11] The luminescence turns to be proportional to E, with a slope of 1. Therefore, the luminescence change obtained on plasmonic substrate in **Figure 3d** and the simulated electromagnetic intensity are consistent in the degree of enhancement. It is the enhanced intensity of the local filed that increases the excited Yb^{3+} ions and induces greater energy transfer from excited Yb^{3+} to Tm^{3+} ions, which results in higher luminescence intensity.

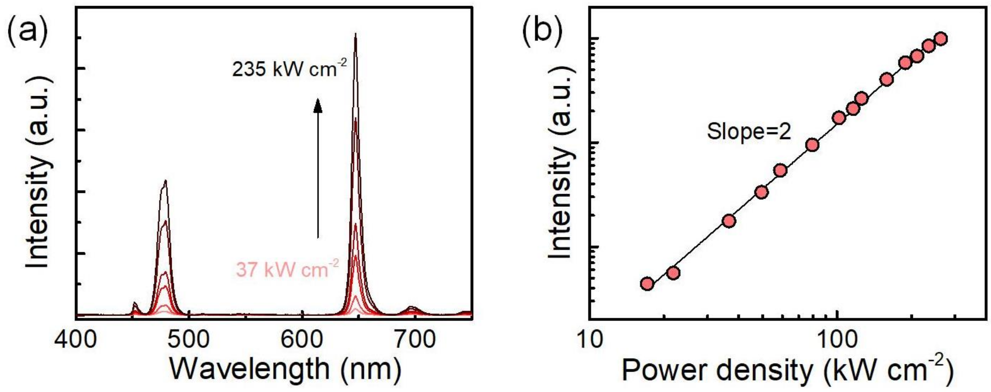

Figure S8. (a) Emission spectra of the UCNPs under different laser power densities (from 37 to 235 kW cm^{-2}). **(b)** Integrated peak intensity at 650 nm as function of the power density.

S10. Effective beam intensity and Brownian motion

In order to take into account the Brownian motion of UCNPs, we implemented the following averaging procedure: We assume that the UCNP wanders in the range $z \in [z_L - w, z_L + w]$, for $z_L > w$, and $z \in [0, z_L + w]$, when $z_L \leq w$, where z_L is the vertical position of the focal point and $2w$ is the width of the wandering interval. Assuming that the UCNP position can be described by a uniform probability distribution, the averaged intensity, effectively experienced by the Brownian UCNP is given by

$$I(z_L, w) = \int_0^\infty dz\, I(z) \Pi_{\mathrm{w}}(z - z_L) \left[\theta(z_L - w) + \frac{2w}{w + z_L} \theta(w - z_L) \right], \qquad (S15)$$

where $\Pi_{\mathrm{w}}(z) = \frac{1}{2w}[\theta(z + w) - \theta(z - w)]$ is a step function of width $2w$ and height $1/2w$, and $\theta(x)$ are Heaviside functions. Note that these are introduced in the expression for $I(z_L, w)$ explicitly to ensure proper normalization of the position distribution function when $z_L < w$ (the vertical distance between the beam focus and the substrate is smaller than the half-width of the position distribution). Normalizing the average intensity over the Gaussian beam in free space, we obtain the intensity enhancement profiles shown in the main text.

S11. Video: Bubble formation due to excessive heating.

https://dauam-my.sharepoint.com/:v:/g/personal/fengchan_zhang_estudiante_uam_es/EYYC7EJnKrRIl0pErDGXhNoBzPdl3obc4CjiU0Pw3ov1Sw?e=MCyE2x

References

1. Gnanasammandhan, M. K.; Idris, N. M.; Bansal, A.; Huang, K.; Zhang, Y. Near-IR photoactivation using mesoporous silica–coated NaYF4:Yb,Er/Tm upconversion nanoparticles. *Nature Protocols* **2016,** 11, (4), 688-713.
2. Tesler, A. B.; Chuntonov, L.; Karakouz, T.; Bendikov, T. A.; Haran, G.; Vaskevich, A.; Rubinstein, I. Tunable Localized Plasmon Transducers Prepared by Thermal Dewetting of Percolated Evaporated Gold Films. *The Journal of Physical Chemistry C* **2011,** 115, (50), 24642-24652.
3. Badilescu, S.; Raju, D.; Bathini, S.; Packirisamy, M. Gold Nano-Island Platforms for Localized Surface Plasmon Resonance Sensing: A Short Review. *Molecules* **2020,** 25, (20), 4661.
4. Jeon, H. B.; Tsalu, P. V.; Ha, J. W. Shape Effect on the Refractive Index Sensitivity at Localized Surface Plasmon Resonance Inflection Points of Single Gold Nanocubes with Vertices. *Scientific Reports* **2019,** 9, (1), 13635.
5. Ozhikandathil, J.; Packirisamy, M. Simulation and Implementation of a Morphology-Tuned Gold Nano-Islands Integrated Plasmonic Sensor. *Sensors* **2014,** 14, (6), 10497-10513.
6. Liu, S.; Lin, L.; Sun, H.-B. Opto-Thermophoretic Manipulation. *ACS Nano* **2021,** 15, (4), 5925-5943.
7. Lin, L.; Peng, X.; Wei, X.; Mao, Z.; Xie, C.; Zheng, Y. Thermophoretic Tweezers for Low-Power and Versatile Manipulation of Biological Cells. *ACS Nano* **2017,** 11, (3), 3147-3154.
8. Domínguez-García, P. Brownian Disks Lab: Simulating time-lapse microscopy experiments for exploring microrheology techniques and colloidal interactions. *Computer Physics Communications* **2020,** 252, 107123.
9. Rakić, A. D.; Djurišić, A. B.; Elazar, J. M.; Majewski, M. L. Optical properties of metallic films for vertical-cavity optoelectronic devices. *Applied Optics* **1998,** 37, (22), 5271-5283.
10. Agate, B.; Brown, C. T. A.; Sibbett, W.; Dholakia, K. Femtosecond optical tweezers for in-situ control of two-photon fluorescence. *Opt. Express* **2004,** 12, (13), 3011-3017.
11. Suyver, J. F.; Aebischer, A.; García-Revilla, S.; Gerner, P.; Güdel, H. U. Anomalous power dependence of sensitized upconversion luminescence. *Physical Review B* **2005,** 71, (12), 125123.
